# The treatment of obesity in children and adolescents: consensus position statement of the Italian society of pediatric endocrinology and diabetology, Italian Society of Pediatrics and Italian Society of Pediatric Surgery

**DOI:** 10.1186/s13052-023-01458-z

**Published:** 2023-06-08

**Authors:** Claudio Maffeis, Francesca Olivieri, Giuliana Valerio, Elvira Verduci, Maria Rosaria Licenziati, Valeria Calcaterra, Gloria Pelizzo, Mariacarolina Salerno, Annamaria Staiano, Sergio Bernasconi, Raffaele Buganza, Antonino Crinò, Nicola Corciulo, Domenico Corica, Francesca Destro, Procolo Di Bonito, Mario Di Pietro, Anna Di Sessa, Luisa deSanctis, Maria Felicia Faienza, Grazia Filannino, Danilo Fintini, Elena Fornari, Roberto Franceschi, Francesca Franco, Adriana Franzese, Lia Franca Giusti, Graziano Grugni, Dario Iafusco, Lorenzo Iughetti, Riccardo Lera, Raffaele Limauro, Alice Maguolo, Valentina Mancioppi, Melania Manco, Emanuele Miraglia Del Giudice, Anita Morandi, Beatrice Moro, Enza Mozzillo, Ivana Rabbone, Paola Peverelli, Barbara Predieri, Salvo Purromuto, Stefano Stagi, Maria Elisabeth Street, Rita Tanas, Gianluca Tornese, Giuseppina Rosaria Umano, Malgorzata Wasniewska

**Affiliations:** 1 Department of Surgery, Dentistry, Pediatrics and Gynecology, Section of Pediatric Diabetes and Metabolism, University of Verona, Verona, Italy; 2 Department of Movement Sciences and Wellbeing, Parthenope University of Naples, Naples, Italy; 3 Deparment of Pediatrics, Department of Health Science, Vittore Buzzi Children’s Hospital, University of Milan, Milan, Italy; 4 Department of Neurosciences, Neuro-Endocrine Diseases and Obesity Unit, Santobono-Pausilipon Children’s Hospital, Naples, Italy; 5 Department of Pediatrics, Vittore Buzzi Children’s Hospital, Milan, Italy; 6 Department of Internal Medicine and Therapeutics, University of Pavia, Pavia, Italy; 7 Department of Pediatric Surgery, Vittore Buzzi Children’s Hospital, Milan, Italy; 8 Department of Traslational Medical Sciences, Section of Pediatrics, University of Naples Federico II, Naples, Italy; 9 Microbiome Reaserch Hub, University of Parma, Parma, Italy; 10 Pediatric Endocrinology Unit, Department of Public Health and Pediatric Sciences, University of Turin, Turin, Italy; 11 Autoimmune Endocrine Diseases Unit, Bambino Gesù Children Hospital, IRCCS, Rome, Italy; 12 Pediatric Unit, Hospital of Gallipoli, Lecce, Italy; 13 Department of Human Pathology of Adulthood and Childhood, University of Messina, Messina, Italy; 14 Department of Internal Medicine, S. Maria Delle Grazie Hospital, Naples, Pozzuoli Italy; 15 Pediatric and Neonatal Unit, Hospital of Teramo and Atri, Teramo, Italy; 16 Department of Woman, Child and General and Specialized Surgery, University of Campania L. Vanvitelli, Naples, Italy; 17 Department of Sciences of Public Health and Pediatrics, University of Turin, Turin, Italy; 18 Department of Precision and Regenerative Medicine and Ionan Area, University of Bari, Bari, Italy; 19 Pediatric Unit Ostuni Hospital, Brindisi, Italy; 20 Refernce Center for Prader Willi Syndrome, Endocrinology and Diabetology Unit, Bambino Gesù Children Hospital, IRCCS, Rome, Italy; 21 Pediatric Unit, S. Chiara Hospital, APSS Trento, Trento, Italy; 22 Pediatric Department, Azienda Sanitaria Universitaria del Friuli Centrale, Hospital of Udine, Udine, Italy; 23 Italian Society for Pediatric Endocrinology and Diabetology (SIEDP), Lucca, Italy; 24 Division of Auxology, Istituto Auxologico Italiano, IRCCS, Verbania, Italy; 25 Department of Medical and Surgical Sciences of Mother, Children and Adults, Pediatric Unit, University of Modena and Reggio Emilia, Modena, Italy; 26 Italian Society for Pediatric Endocrinology and Diabetology (SIEDP), Alessandria, Italy; 27 Local Health Unit Napoli, 3 Sud, Naples, Italy; 28 Research Area for Multifactorial Diseases, Children’s Hospital Bambino Gesù, Rome, Italy; 29 UOSD Diabetology, Complesso Ai Colli, AULSS 6 Euganea, Padua, Italy; 30 Division of Pediatrics, Department of Health Sciences, University of Piemonte Orientale, Novara, Italy; 31 Department of Pediatrics and Gynecology, Hospital of Belluno, Belluno, Italy; 32 Pediatric Obesity Unit, ASP of Ragusa, Ragusa, Italy; 33 Department of Health Sciences, University of Florence, Meyer Children’s Hospital IRCCS, Florence, Italy; 34 Department of Medicine and Surgery, Unit of Paediatrics, University of Parma, University Hospital of Parma, Parma, Italy; 35 Italian Society for Pediatric Endocrinology and Diabetology (SIEDP), Ferrara, Italy; 36 Institute for Maternal and Child Health, IRCCS “Burlo Garofolo”, Trieste, Italy

**Keywords:** Children, Adolescents, Obesity, Treatment, Nutrition, Physical activity, Cognitive and family-based behavior therapy, Drug, Bariatric surgery

## Abstract

This Position Statement updates the different components of the therapy of obesity (lifestyle intervention, drugs, and surgery) in children and adolescents, previously reported in the consensus position statement on pediatric obesity of the Italian Society of Pediatric Endocrinology and Diabetology and the Italian Society of Pediatrics. Lifestyle intervention is the first step of treatment. In children older than 12 years, pharmacotherapy is the second step, and bariatric surgery is the third one, in selected cases. Novelties are available in the field of the medical treatment of obesity. In particular, new drugs demonstrated their efficacy and safety and have been approved in adolescents. Moreover, several randomized control trials with other drugs are in process and it is likely that some of them will become available in the future. The increase of the portfolio of treatment options for obesity in children and adolescents is promising for a more effective treatment of this disorder.

## Introduction

The treatment of childhood and adolescent obesity is urgent due to at least three main factors: *i.* the dramatic epidemiological impact of obesity, which is continuously increasing in all the world; *ii.* the association of obesity with metabolic [impaired glucose tolerance and diabetes, hypertension, dyslipidemia, non-alcoholic fatty liver disease (NAFLD), etc.] and non-metabolic (weight stigma, teasing, bullying, victimization, low self-esteem, depression, body -image disturbances, eating, respiratory and orthopedic disorders, etc.) disorders; *iii.* the reduction of life expectancy in youth with obesity [1–3].

Overweight and obesity from the earliest stages of life are risk factors for the persistence of obesity and more than 60% of overweight, prepubertal children maintain the extra weight into adulthood. Exposure to excess adiposity, especially if prolonged, is associated with cardio-metabolic risk factors and the consequent development of cardiometabolic comorbidities such as hypertension, dyslipidemia, type 2 diabetes and NAFLD, which have an important impact on the overall mortality of the population [4]. Moreover, functional deficits, with a severe restriction of motor skills and psycho-social unease are common in youth with severe obesity and affect the quality of life, leading to true disability [5].

The National Institute for Health and Care Excellence suggests lifestyle intervention as the primary approach to managing obesity [3]. However, available data suggest that therapeutic interventions for childhood and adolescent obesity have shown modest efficacy, especially in the long term [6, 7], and relapse is fairly common. In recent years, bariatric surgery and drug therapy showed some encouraging results on substantial and lasting weight loss and contribute to the increase of the treatment portfolio available for the clinician [8].

Therefore, on the basis of this evidence, the Italian Society of Pediatric Endocrinology and Diabetology, the Italian Society of Pediatrics and the Italian Society of Pediatric Surgery made this consensus on the treatment of obesity in children and adolescents, with the purpose to provide an update on available treatments for obesity in pediatrics. This consensus does not cover prevention and the treatment of obesity-comorbidities.

## Methods

An expert group on obesity in children and adolescents was involved in document processing. The definition of obesity is based on body mass index (BMI) > 99^th^ centile for 2–5-year-old children and > 97^th^ centile for children older than 5 years till 18 years, using World Health Organization (WHO) BMI reference [9, 10]. A principal coordinator was identified for each of the five areas of treatment of childhood and adolescents: 1. Nutrition, 2. Exercise and Physical activity, 3. Cognitive and family-based behavior therapy, 4. Medical treatments, 5. Bariatric Surgery.

For each topic, a systematic review of the literature was conducted using database PubMed with MeSH terms or descriptors, limited to patients 0–18 years old and from May 2016 to November 2022.

Scientific articles, systematic reviews, meta-analysis, consensus, recommendations, international and national guidelines published on pediatric obesity even prior to 2016 were considered and deemed useful to the Consensus. The level of evidence (LOE) and the grade of recommendations were established in accordance with the National Manual of Guidelines [11].

Each working group prepared a preliminary draft reporting the LOE for each specific recommendation, followed by a brief description of the scientific evidence in support, epidemiological data, and any notes deemed as useful. The final document was sent on December 2022 to all the extensors and members of the Pediatric Obesity Study Group of the Italian Society for Pediatric Endocrinology and Diabetology and approved on January 2023 in its definitive form. The literature search was updated before preparing the final draft; no additional relevant publications were identified that might have required a change in the statements.

## Targets of treatment

The treatment of obesity, i.e., the excessive accumulation of fat in the body, should be based on an action on the specific causes of this condition. In less than 10% of cases, obesity is secondary to other diseases, such as syndromes, monogenic obesity, endocrine disorders, drugs, cancer, psychological and psychiatric disorders. The treatment of the primary disease is therefore the main target of therapy.

In more than 90% of cases, the specific cause of obesity is not known and an etiologic based treatment is not possible. Therefore, a reasonable target of treatment is to maintain a chronic negative energy balance to reduce the fat mass level. To achieve and maintain this objective, it is necessary to act on the factors contributing to regulating food intake and energy expenditure. A crucial issue is to avoid any kind of stigmatization of weight excess, that requests a careful sensitivization and training of all professionals involved in the care procedure.

At present, three treatment options for children and adolescents with obesity are available: lifestyle intervention, pharmacotherapy, and bariatric surgery.

The purpose of this Consensus is to provide an overview of the current treatment options for pediatric severe obesity in different age groups.

## Lifestyle intervention

Multidisciplinary lifestyle intervention programs that focus on nutrition, physical activity, and behavioral change are the first and most frequently applied treatment options for obesity.

In particular, lifestyle intervention was effective on weight loss in children with obesity, but almost no effect was found in adolescents with severe obesity [12]. Danielsson et al*.* reported that the BMI z-score was reduced at least 0.5 units during a three-year intervention in 58% of the 6–9 years old children with severe obesity, but in only the 2% of the adolescents (14–16 years old) [13], underlining the importance to start treatment for severe obesity at an early age. Possible reasons for the low response of adolescents to lifestyle interventions are the decline in parental influence during adolescence, which was demonstrated to play a key role in the treatment process [14], and the reduced adherence to these interventions as age increases [15].

However, the evidence that lifestyle intervention is effective in treating comorbidities that often accompany pediatric severe obesity further justifies its use as the first step in the treatment program even if clinically meaningful weight loss is difficult to achieve in youth with severe obesity, especially adolescents [16, 17].

## Nutrition

Nutrition is an important target of treatment: dietary components have a modest but sustained impact on reducing total energy intake and improving intakes of specific food groups in youths with overweight or obesity that can be deficient [18].

Different diet interventions have been tested in the treatment of obesity, including modification of macronutrient composition, implementing different dietary patterns, and changing meal timing.

Ideally, in the clinical practice, a child nutrition plan should be individualized based on age, food preferences, cultural preferences, family and personal lifestyle, and concurrent medical diseases.

A recent review of 28 clinical practice guidelines, consensus and position statements concluded that dietary approaches based on caloric restriction are recommended to achieve weight loss although no evidence of the long term durability of the results obtained is available [19, 20]. However, guidance on the management of severe obesity (BMI > 99^th^ centile for children older than 5 years) using dietary approaches is currently unavailable.

Few guidelines recommend intensive dietary approaches for the management of obesity with related comorbidities or severe obesity, such as very low-energy diet (VLED) and very low-carbohydrate diets (VLCD).

## Non-restrictive approach

This dietary model does not consider a given calorie intake or a composition of nutrients, rather it focuses on the consumption of low-fat and high-nutrient-dense foods (e.g. reducing fruit juice consumption and increasing fresh fruit consumption). It has been suggested that, to provide advice targeted towards specific food components (for example the reduction of the consumption of carbonated drinks and sugary foods or foods with a high caloric density), is more effective in weight control than providing advice on healthy eating. Supporting these long-term dietary changes has the potential to improve energy balance and decrease the BMI z-score in children and adolescents aged 2–18 years [21–23].

The Italian Guidelines for healthy and proper nutrition, issued by Council for Agricultural Research and Analysis of Agricultural Economics of Italy (CREA) in 2019, report both the portions by age and the weekly recommended daily consumption frequencies.

This approach begins with the assessment of the child’s and family’s eating habits, through the detection of the composition of meals, portions, frequency of food intake, food preferences or aversions, the eating habits of the family. The 3-day food diary or the diet history are good tools for assessing dietary intake, providing adequate instruction for filling in the diary. (LOE VI-A)

Several lifestyle changes must go hand in hand with non-restrictive approach, such as paying attention to food consumption while screen watching. In fact, a systematic analysis has shown the positive association between watching television in the 1–18 age group and the consumption of snacks, sugary carbonated drinks and foods with a high calorie density [24].

Furthermore, it is important to educate the patient to consume meals and snacks on appropriate occasions of consumption. In particular, a frequency of 5 meals per day, has been associated with a lower risk of developing obesity in children and adolescents [25]. On the contrary, nighttime consumption of snacks has been associated with an increased risk of obesity in children with low levels of physical activity [26]. However, randomized clinical trials (RCTs) are necessary for confirming these results. (LOE III-A)

### Traffic light diets (modified Traffic light diets)

The Traffic Light diet is a calorie-controlled approach in which foods in each category are color-coded according to their calorie density per serving: green for low-calorie foods that can be eaten freely; yellow for moderate-calorie foods that can be eaten occasionally; and red for highcalorie foods that should be eaten rarely. Children and adolescents with obesity following this diet showed an improvement in the anthropometric parameters of obesity (BMI z-score, waist circumference, fat mass), with maintenance of the result even at 1 year follow-up [27]. (LOE I-B)

### Diets with low glycemic index and low glycemic load

The low carbohydrate (LC) diet limits carbohydrate (CHO) intake to no more than 60 g/day, and the reduced glycemic load (RGL) diet restricts intake of rapidly absorbed carbohydrates [28].

The glycemic index (GI) is defined as the ratio of the area under the blood glucose curve measured two hours after consuming 50 g of test carbohydrates and the area under the blood glucose curve obtained by 50 g of glucose or white bread. The term glycemic load (GL) was introduced to quantify the overall glycemic effect of the food compared to amount of CHO contained in the serving (usually 100 g). Diets that consider the GI and the GL imply an evaluation not only quantitative of the selected foods, but also qualitative (for example, semolina pasta 82.8 g CHO, whole meal semolina pasta CHO 66.2 g). The patients are instructed to distinguish: “high glycemic index” foods such as sugary drinks, refined baked goods, candies and sweets, white bread and potatoes (GI ≥ 70), “intermediate glycemic index” foods such as pasta, corn and dried fruit (GI = 56–69) and “low glycemic index” foods such as fruit, non-starchy vegetables, 100% whole grains, meat, fish and poultry (GI ≤ 55). While the last category can be consumed without restriction, the intermediate and high GI categories must be introduced respectively less frequently and occasionally during the week [28]. There are no specific restrictions on energy or fat intake.

Diets using modified CHO intake have resulted in improved weight status even in the medium-long term period (12 months) in children and adolescents [28].

Normal calorie diets with a low GI and/or low GL can be useful in children and adolescents with obesity and glucose metabolism alterations, with positive impact in fasting blood sugar, fasting insulin and homeostasis model assessment (HOMA)-index [28, 29]. (LOE I-A)

### Mindful eating

Recently, new strategies have been proposed for the treatment of obesity focused on the factors that influence patients’ abilities to acquire behavioral changes necessary to follow diet therapy. Mindful Eating Approaches aim to make the subjects aware of their food choices, develop attention to one’s physical and not psychological fullness, respect the stimuli of satiety and finally consume healthy foods precisely in response to such stimuli [30]. The primary objective is therefore not weight loss but the ability to perceive and respond appropriately to one’s own stimuli appetite/satiety. Despite the growing interest in this treatment, studies in children with obesity are currently limited and aimed primarily at adolescents. A RCT demonstrated the greatest efficacy of this type of treatment, compared to a control group with traditional nutritional advice, in reducing BMI in adolescent girls (14–18 years) in the short-term (10 weeks) [31]. In addition, a recent study on adolescents (12–17 years) at risk of obesity has shown how the Mindful Eating approach is more effective than standard health education in reducing food reward sensitivity or stress-eating after six months [32]. Similarly, a study targeting adolescents living with obesity (14–18 years) and their families showed a reduction in distraction during the meal and an increased food awareness following a Mindful Eating approach compared to standard nutritional counseling [33].

This approach has also been reported to be effective to prevent childhood obesity [30]. (LOE II-C)

## Balanced low calorie diets

Balanced low-calorie diets are effective in promoting weight loss in children [27, 34, 35]. The diets most frequently reported include a 30% reduction in the average daily caloric intake, measured through a 3-day food diary or Food Frequency Questionnaire (FFQ), or a 15% reduction compared to the energy requirement estimated through predictive equations for gender and age [27]. This dietary approach combined with changes in physical activity level has been reported to be effective in treating children and adolescents with obesity [36]. After 3 months of intervention, significant reduction of BMI has been observed compared to the control group (− 1.7 ± 1.1 kg/m^2^ vs − 0.2 ± 1.0 kg/m^2^), and the difference in BMI between the two groups was maintained even at 1 year of follow up (− 1.7 ± 2.3 kg/m^2^ vs 0.6 ± 0.9 kg/m^2^). Long-term efficacy was demonstrated even without changes in the level of physical activity in a RCT targeting children with overweight or obesity aged 7–12 years [28].

This intervention involves the formulation of a food plan, periodically re-evaluated during the follow-up in relation to the nutritional needs of the growing child or adolescent, which respects a balanced distribution of macronutrients suggested by Reference Intake of nutrients and energy for Italian Population (LARN) (proteins: 10–15% of daily energy, between 0.9–1 g/kg/day depending on age; CHO: 45–60% of daily energy, simple sugars < 15% of total calories; and lipids: 35–40% of total calories in the 1–3 year-old children and from 4 years 20–35% of daily energy, of which saturated fatty acids < 10% of daily energy) [37]. (LOE I-B)

### Low-calorie high-protein diets

The high-protein diets are characterized by a protein intake increased up to 19–30% of the daily energy intake, with a reduction of CHO intake to 35–50% of total energy, while lipid intake remains in the range of 25–35% of total energy.

A meta-analysis of six trials evaluated the impact of a high-protein diet versus a balanced low-calorie diet on the outcome of obesity indices (primarily assessed as BMI, BMI z-score, and/or body composition) in a group of 6–18 years old subjects [35]. No differences of the level of overweight and metabolic parameters (glycemia, insulin sensitivity and blood pressure) between the two treatments have been found, so that there is no evidence to recommend high-protein low-calorie diets for the treatment of obesity in school age and adolescence. (LOE I-D)

## Hyperlipidic low-calorie diets

High-fat nutritional approaches have been poorly investigated in the pediatric population with obesity. A study in a group of a 9–14-year-old girls compared a high-fat diet (42% CHO, 40% fat and < 10% saturated fat, ~ 18% protein) with a normal fat diet (55% CHO, 27% fat with < 10% saturated fat, ~ 18% protein). At 11-weeks, the change of BMI was not different between the two groups [38]. (LOE II-D)

### Very low-energy diet or very low-carbohydrate diet

Diets with a low or very low energy content (from 600–800 up to 1,200 kcal/day) or low carbohydrate content (< 20 g CHO/day or 10–20% of total daily energy) have been investigated in last years in children and adolescents with obesity. These approaches have a limited duration, generally between 8 and 20 weeks.

A 2014 systematic review demonstrated improvement in BMI, BMI z-score or body composition following the adoption of this regimen for a short period of time (1–6 months duration) in children and adolescent aged 6–18 years. However, there is no significant difference in weight loss between the highly hypocaloric dietary regimen and a balanced low-calorie dietary approach (50–60% CHO and < 33% fat or less than 40 g/day) after two years [35].

A second systematic review and meta-analysis in 2019 evaluated the effects of low-calorie diets (600–800 kcal/day or < 50% of energy requirement) in 5–18-year obese subjects, demonstrating the effectiveness of this treatment in reducing weight and BMI values (but BMI z-scores were not reported) in the short term (6 months), although a comparison with a standard dietary approach was not provided. Six studies included adolescent and no studies were conducted in children [39]. The authors concluded that this dietary treatment may be particularly useful for achieving weight loss, in particular in adolescents with comorbidities related to obesity or eligible for bariatric surgery. Furthermore, the authors stressed the importance of adopting this treatment in a hospital setting or in centers specialized in the treatment of pediatric obesity and under medical supervision [39]. (LOE I-C)

However, the compliance of these diets is low and the long-term outcomes is comparable to that of the balanced low-calorie diets. Moreover, it is not recommended to undertake low-calorie diets especially in school-age children, since. The safety of these dietary approaches on the growing subject has not yet been demonstrated. (LOE I-D)

## Dietary patterns

### Mediterranean diet

The Mediterranean diet (MD) is characterized by a high intake of vegetables, fruit, oily nuts, whole meal cereals, legumes and olive oil, as well as a moderate consumption of fish and poultry and a low intake of sweets, red meat and dairy products [40]. It promotes increased consumption of dietary fiber, antioxidants and long-chain fatty acids.

Dietary models based on the principles of the MD can be used as a normocaloric approach for the treatment of obesity in children and adolescents. Despite the high number of studies that have investigated the effect of adherence to the MD on obesity, methodological limitations in most of the intervention studies, such as non-inclusion of a control group or lack of analysis considering confounding factors, lead to the conclusion that there is limited evidence of a beneficial effect in following a MD to obtain weight loss in children with obesity [41–47]. This recommendation is mainly based on expert opinion [41, 48]. (LOE VI-A)

### DASH diet

The DASH (Dietary Approach to Stop Hypertension) diet is a dietary pattern that advocates the daily introduction of at least 8 portions of fruit and vegetables, two or three daily portions of dairy products or low-fat dairy products and a sodium consumption ≤ 2.4 g/day.

The nutritional indications also include a reduced consumption of foods with a high fat and cholesterol content, such as red meat, whose portions must be reduced to two or less (per day). The indications aim to ensure a balanced macronutrients distribution, without a necessary energy-restriction (53–58% CHO, 15–18% protein and 26–30% fat) [49].

This nutritional approach has proven effective in improving several comorbidities associated with obesity and metabolic syndrome in children and adolescents. In particular cohort studies and cross-sectional studies highlight an association between the DASH diet and beneficial effects on blood pressure, overweight and obesity in 11–18 year-old adolescents [50], although these results were not obtained in intervention studies [49]. (LOE III-C)

## Exclusionary diets

### Vegetarian and vegan diet

These two dietary patterns are characterized by the exclusion of meat and seafood from the diet. In addition, the vegan diet excludes “products of animal origin”, such as milk, dairy products, eggs and honey, which are allowed in the vegetarian model.

These eating patterns are effective in the treatment of obesity in adulthood [51, 52]. On the contrary, studies evaluating the effectiveness of this dietary model for the treatment of the obese pediatric patient are currently lacking. There is therefore no evidence to recommend vegetarian and vegan diets for the treatment of childhood obesity [48]. (LOE VI-D)

## Physical activity

Physical activity, defined as “any movement of the body produced by skeletal muscles that requires energy expenditure”, plays an important role in the treatment of obesity since it contributes to increasing energy expenditure and obtaining weight loss without significant variations of fat free mass (FFM) [53].

Fat free mass is the strongest predictor of the resting metabolic rate and its reduction have significant implications in the long-term weight management. Interventions based on supervised and structured programs of physical activity, such as aerobic and/or resistance exercise combined to diet may counteract the negative effects on FFM compared to interventions based on diet only [54, 55].

Programs encourage to achieve the recommended levels of physical activity through an increase of both organized and non-organized physical activities. In order to achieve optimal outcomes, recommendations about type and amount of physical activity should be age specific and should consider the extent to which the severity of obesity impairs the components of physical fitness, the musculoskeletal system and the quality of life. Children and adolescents with obesity have lower coordination, balance, speed, agility and fine and gross motor skills compared to their healthy weight peers [56]; this hinders the adherence to the physical activity goals and sustains a vicious circle of inactivity and weight excess. In addition, poor motor skills expose children to bullying victimization and contribute to low self-esteem and poor quality of life [57]. For these reasons, increasing physical activity and exercise can be difficult in children with obesity and an adapted physical activity intervention is needed for a successful outcome.

Ideally, exercise promotion and treatment should be included in the setting of multidisciplinary teams, composed of exercise physiologists or activity specialists trained to manage the physical barriers of children with obesity, working in concert with other professionals. Realistically, these experts are lacking in the multidisciplinary teams in most countries of the WHO European Region [58].

## Young children (3–5 years of age)

The rise in childhood obesity in this period is mostly driven by unbalanced energy intake rather than decline in physical activity [59]. Progression in many of the fundamental motor skills, such as running, jumping, throwing, and catching, typical of this developmental stage, may be negatively influenced by weight excess. Combination of increased physical activity and reduced sedentary behaviors should be promoted in preschool children with obesity as an essential component of a healthy lifestyle, improvement of fundamental movement skills, motor coordination activities and fitness [60, 61]

Pre-schoolers, independently from BMI, should spend at least 180 min in a variety of types of physical activity at any intensity, of which at least 60 min is moderate/vigorous physical activity, distributed throughout the day. In addition, they should not be restrained for more than one hour at a time and should limit the screen time to less than one hour/day [62, 63].

The physical activity goals may be achieved through promoting free play, with emphasis on safe, enjoyable and supervised motor tasks [64]. (LOE VI-A)

## Children and adolescents (6–17 years of age)

At this age, physical inactivity and sedentary behaviors are significant contributors to the development of pediatric obesity and increase the cardiometabolic risk. Both inactivity and body fat excess significantly impair several components of the health related physical fitness, such as aerobic and muscular fitness [65, 66].

Both components have been associated to a healthier cardiometabolic profile [67–69] and may attenuate the adverse effects of obesity on insulin-resistance and cardiometabolic risk [70]. Exercise in children and adolescents with obesity can improve anthropometric (body mass, BMI, central obesity, fat mass) cardiovascular (triglycerides, fasting glucose, fasting insulin) parameters, and cardiorespiratory fitness [71].

Regarding the type of exercise, either aerobic or combined aerobic and strength training have been associated with significant and clinically improvements in BMI, fat mass and fat mass percent in children and adolescents with overweight and obesity [72].

Therefore, physical activity and exercise at this age should be designed to improve both components of physical fitness, without overlooking fundamental movements and motor coordination skills.

Children and adolescents should accumulate at least 60 min per day of moderate-vigorous physical activity, involving a variety of aerobic activities based on gross motor skills (walking, cycling, swimming); activities that promote flexibility and muscle strength should be performed at least three days per week [73]. As in young children the proposed activities should consider the ease and enjoyment of moving and playing.

Structured exercise programs based on aerobic and/or resistance training or sports participation in gym or sport facilities can be performed at moderate intensity, gradually increasing the frequency, intensity and duration over time.

A research gap is still represented by establishing the optimal exercise dose for promoting weight loss in children and adolescents living with overweight or obesity. According to a recent meta-regression analysis that included aerobic exercise and strength training interventions, a positive linear association between exercise dose and weight loss was found without identifying the best dose. In most studies the exercise prescription dose was below the recommended amount of physical activity (roughly 150 min of moderate activity per week).

In addition, the daily physical activity outside the intervention was not considered. This issue is not of secondary importance, since it is still questioned whether high amount of exercise can negatively influence the total weekly physical activity [74].

Conversely, there is much evidence about the amount of physical activity able to produce health outcomes. Physical exercise programs lasting 4–12 weeks, or sessions of 60 min each, or involving a total exercise time of at least 1,500 min were effective in improving cardiometabolic and vascular parameters (lipids, fasting glucose, fasting insulin, homeostasis model assessment of insulin resistance [HOMA-IR], intrahepatic fat, systolic blood pressure, and carotid intima-media thickness) in children with obesity [75].

As part of behavioral approaches, sedentary activity should be reduced, particularly the amount of recreational screen time to a minimum of two hours daily [73]. (LOE I-A for excercise, LOE I-B for sedentary reduction)

### Cognitive and family-behavioral therapy

In the treatment of obesity, a substantial, feasible, lifestyle change is recommended through an integrated approach to diet, exercise and strategies aimed at changed behavior. (LOE I-A)

To get better adherence to diet and exercise programs, the main strategies of intervention proposed in the literature are behavioral therapy (BT), cognitive behavioral therapy (CBT) and therapeutic education of the patient (ETP) [76]. (LOE VI-A)

Furthermore, recent studies show that strategies aimed at behavioral change are associated with a greater probability of long-term maintenance of the results obtained through calorie restriction and increased physical activity [77]. (LOE I-A)

Behavioral therapy aims at modifying dysfunctional eating and motor behaviors without affect the personality of the subject [76]. (LOE VI-A)

Behavioral therapy uses behavioral techniques, which can also be used in combination such as: self-monitoring, goal setting, problem solving, social support, stress management, stimulus control, alternative behaviors, environmental control parental monitoring, parental modeling, positive reinforcement and fragmented techniques [76]. (LOE VI-B)

Cognitive behavioral therapy aims to change the thoughts that induce dysfunctional behavior and hinder change. Cognitive strategies in addition to the behavioral ones are more effective than BT alone, however, they are less applicable to pre-adolescents and entire families [76]. (LOE VI-A)

The ETP’s purpose is to know the disease, manage the disease and the therapy, as well as, prevent avoidable complications. The tools of the ETP adapted to obesity in childhood are: therapeutic relationship, reflective and proactive listening, family approach, modeling, counseling motivational, therapeutic storytelling, positive reinforcement, small steps, bargaining on goals [76]. (LOE VI-B)

Recent systematic reviews, published on Cochrane, have assessed the importance of using such cognitive behavioral approaches on weight loss in pediatric-adolescent-aged subjects (0–6 years, 6–11 years and 12–17 years) with different levels of validity.

#### 0–5 years

Colquitt et al. [78] reported that a multidisciplinary approach related to diet and physical activity integrated to a strategy aimed at lifestyle change in young children is an effective treatment option compared to diet and physical activity alone. The multicomponent intervention promoted an improvement of BMI z-score, waist circumference, health-related quality of life [79–82]. Moreover, other positive outcomes have been reported, such as an increase of daily vigorous physical activity, a reduction of sugary drinks intake and time of video exposure, and an increase of parents’ beliefs, attitudes and practices regarding feeding children [80–83]. (LOE I-B)

#### 6–11 years

Mead et al*.* [20], analyzed the same primary and secondary outcomes in patients treated with diet, physical activity and behavioral therapy compared to those who followed diet and physical activity alone, selecting RCT studies (range of ages 6 to 11).

Cognitive and/or behavioral therapy in addition to treatment was found to improve adherence to short- and long-term therapy. In particular Epstein et al. [84] evaluated that the addition of problem-solving exercises with or without parental involvement in 6–11 years old is associated with improvement of primary outcomes (BMI and BMI z-score). Taveras et al*.* [85] found that a family and individual coaching as clinical support intervention is associated with more easily maintained weight loss, even in the long term. The primary results showed that interventions that change behavior recorded lower values of BMI, BMI z-score and body weight compared to the absence of treatment, at the short-term follow-up. (LOE I-B)

#### 12–17 years

Al-Khudairy L et al*.* [86] regarding the age range from 12 to 17 years, found that multidisciplinary interventions with the combination of diet, exercise and behavior modification further reduced BMI and weight of adolescents with overweight and obesity, compared to those who did not receive any treatment (no specific food program and maintained in follow up) or only with only a dietary approach and not supported by psychological therapy. (LOE I-B)

The National Institute for Health and Care Excellence (NICE) guidelines state that “multicomponent interventions are the treatment of choice for obesity and these should include the behavioral component” [87]. Furthermore, a systematic review of 37 randomized trials comparing patients treated with BT versus non-behavioral approaches [88], highlighted greater efficacy in cases than in controls (with a difference weighted mean of − 2.8 kg in the treated group after 12-months). (LOE I-A)

A review of 266 articles related to healthy eating interventions using behavior change theory was recently published [89]. In one of the articles reported, Kelly et al*.* [90], highlighted that many people may lack knowledge and skills to follow a proper diet. Therefore, dietary education alone may not be sufficient to guide a change in nutritional behavior and dieticians/nutritionists should perform an important role in changing nutritional behavior and the role of nutrition education. [91]

The most common theories of behavior change identified in the published studies are: the social cognitive theory [92], the most used according to Rigby et al*.* [93]; the theory of planned behavior [94], the trans-theoretical model [95], the model of health knowledge [96] and the theory of self-determination [97]. (LOE I-A)

A recent RCT study which aimed to develop a multidisciplinary lifestyle intervention program for children and adolescents with moderate to severe obesity, evaluated the additional effects on outcomes compared to traditional treatments [98]. Overall, 103 participants in the 85^th^ percentile of age and sex-specific BMI were selected and divided into groups that received 16 weeks of routine care or multidisciplinary interventions.

This study showed that the intervention had positive effects on body composition (BMI and BMI z-score), cardiometabolic risk markers and in maintenance of weight loss at the 16-week follow-up [99]. (LOE I-A)

## Medical treatment

Pharmacotherapy is the next step in obesity management for patients who fail to achieve their weight loss goals with lifestyle modification therapy alone [100]. Indications for pharmacotherapy in pediatric obesity are patients aged 12 years or older and having BMI ≥ 95^th^ percentile with weight-related comorbidities or BMI ≥ 120% of the 95^th^ percentile regardless of comorbidities, who have not properly responded to lifestyle modification [101]. Management of drugs should be done in third level centers [102].

Weight loss medications, although effective, have low popularity, high costs (usually not covered by the national health care systems), and concerns regarding their safety still persist due to historical problems associated with their use [103]. Studies report that between 3 and 44% of patients taking weight loss medications may experience side effects [104]. However, recent data on weight loss medications show promising results in adolescents [105, 106].

In the last few years, more drugs have become available for the treatment of youth with obesity, but options approved are limited. The Food and Drug Administration (FDA) has approved once-daily liraglutide (3.0 mg), orlistat (120 mg), and phentermine–topiramate (7.5 mg of phentermine with 46 mg of topiramate or 15 mg of phentermine with 92 mg of topiramate) for adolescents at least 12 years of age [107–109]; only liraglutide is approved by the European Medicines Agency (EMA) [110, 111].

*Orlistat* (tetra-hydro-lipstinate) is FDA-approved for long-term treatment of obesity in adolescents aged ≥ 12 years. The medication has not been approved for use by the EMA. By inhibiting pancreatic and gastric lipase, it decreases lipid absorption [111].

In the largest pediatric trial, children were randomized to either orlistat 120 mg or placebo three times daily over 52 weeks. At the end of the trial, BMI had decreased by 0.55 kg/m^2^ with orlistat and increased by 0.31 kg/m^2^ with placebo. Furthermore, 26.5% of children in the orlistat group had a 5% or greater decrease in BMI compared with 15.7% of those in the placebo group [112]. Of note, only 64% of participants in the control group and 65% in the orlistat group completed the trial. The most common adverse events in the orlistat group were gastrointestinal-related, generally of mild to moderate intensity [113]. (LOE II-B)

*Phentermine* is approved in the United States for adolescents > 16 years of age for up to 12 weeks, but not in Europe. Through reduction of norepinephrine reuptake, it stimulates pro-opiomelanocortin (POMC) neurons in hypothalamus [114]; additionally, it improves appetite inhibitory control on prefrontal cortex by acting on serotonin and dopamine reuptake [115, 116]. Weight loss is small to moderate. This drug may cause anxiety, tremors, slightly increased blood pressure. The main studies evaluating phentermine for the treatment of obesity in adolescents showed scarce safety and efficacy data reported [117]. Increased blood pressure and heart rate are common side effects [118]. The paucity of long-term data for phentermine, along with its short-term use indication represents an important limitation, considering the need for chronic treatment of obesity [119]. (LOE IV-B)

*Topiramate* is FDA-approved for neurological disorders and also, in combination with phentermine, is indicated for obesity in patients ≥ 12 years of age [120]. Topiramate blocks neuronal sodium channels, antagonizes glutamate receptors, inhibits carbonic anhydrase, and is thought to suppress appetite through an increase in gamma-aminobutyric acid (GABA) activity. Topiramate use for 6 months has been observed to correlate with a reduction in BMI between 2 and 4.9%. Its use for obesity in subjects under 18 years of age is off-label [120, 121]. Randomized controlled clinical trials are needed to examine efficacy and safety of topiramate for severe obesity in adolescents [121]. (LOE III-C)

In addition to the anti-obesity drugs, metformin is approved by the US FDA to treat type 2 diabetes in children aged over 10 years.

It is delivered orally and acts on reducing glucose levels by inhibiting hepatic gluconeogenesis and promoting intestinal absorption of glucose [122, 123]. Even though metformin does not often result in significant body weight loss, it appears to prevent or delay alteration of glucose homeostasis in children at high risk of developing type 2 diabetes mellitus [124]. Furthermore, metformin has also an off-label use to achieve weight loss in children. Masarwa et al*.* reported metformin efficacy in a systematic review of RCT studies in children and adolescents. They demonstrated that metformin use offers modest benefits in reducing BMI in subjects with obesity [125]. Specifically, when BMI z-score variable was studied by evaluating seven RCTs, it was observed that metformin consistently produced a decrease in this parameter compared with placebo, with more evident results in children and adolescents with nonalcoholic fatty liver disease [100, 125]. In addition, metformin has been shown to improve cardiovascular risk profile and inflammatory biomarkers in obese children and adolescents [126, 127]. Side effects are usually gastrointestinal, including bloating, diarrhea, and flatus, and are not reported as serious, with a discontinuation rate due to adverse events < 5% [118]. (LOE I-B)

Glucagon-like peptide 1 (GLP-1) receptor agonists are incretins that enhance insulin secretion and improve satiety by slowing gastric emptying and acting on arcuate nucleus of hypothalamus, limbic/reward system in amygdala, and the cortex [128–130]. A recent meta-analysis indicates that GLP-1 receptor agonists are safe and effective in modestly reducing weight, BMI, glycated hemoglobin A1c, and systolic blood pressure in children and adolescents with obesity in a clinical setting. Apart from increased rates of minor gastrointestinal -related symptoms such as nausea, no serious adverse events were noted [131].

*Liraglutide* was approved for obesity treatment in children aged 12-17 years (weight > 60 kg and BMI of > 30 kg/m^2^ in accordance with international standards / ≥ 95^th^ percentile) from both the FDA and the EMA. The efficacy and safety of liraglutide in adolescents were demonstrated in a randomized, double-blind trial, enrolling 251 adolescents (ages 12-18 years) with obesity and a poor response to lifestyle therapy alone. The trial consisted of a 56-week treatment period and a 26-week follow-up period. Participants were randomly assigned (1:1) to receive either liraglutide (3.0 mg) or placebo subcutaneously once daily, in addition to lifestyle therapy liraglutide was superior to placebo in reducing BMI standard-deviation score from baseline to the 56^th^ week of treatment (estimated difference, − 0.22; 95% CI [Confidence interval], − 0.37 to – 0.08). A reduction in BMI of at least 5% was observed in 43.3% of participants in the liraglutide group and 18.7% participants in the placebo group, and a reduction in BMI of at least 10% was observed in 26.1 and 8.1%, respectively. A greater reduction was observed with liraglutide than with placebo for BMI (estimated difference, − 4.64 percentage points) and for body weight (estimated difference, − 4.50 kg). At week 56, there was no substantial difference between treatment groups in glycemic and cardiometabolic variables or in overall weight-related quality of life [132]. The adverse events most frequently reported in the liraglutide group were gastrointestinal events including nausea, vomiting, and diarrhea, which represented the main cause of discontinuation of the trial treatment. In summary, in adolescents with obesity, liraglutide 3.0 g as an adjunct to lifestyle therapy led to a greater reduction in the BMI than placebo [132]. (LOE I-A)

*Exenatide* is used off label under 18 years of age in obesity, in presence of diabetes, and/or in hypothalamic or syndromic obesity, for example Prader Willi syndrome [100, 111]. In a previous randomized, double-blinded, parallel, placebo-controlled clinical trial provides that exenatide XR is feasible, generally well tolerated, and leads to reduction, albeit modest, in BMI z-score and a significant improvement in glucose tolerance in adolescents with obesity [133]. In a recent randomized, double-blind trial exenatide partly mitigated the BMI rebound in adolescents who achieved weigh loss with dietary intervention (meal replacement therapy run in phase up to 8 weeks) [134]. (LOE II-B)

*Semaglutide* is another GLP-1 receptor agonist, recently approved by FDA in pediatric patients 12 years of age and older. In the recent Semaglutide Treatment Effect in People with Obesity (STEP) TEENS trial, a double-blind, parallel-group, randomized, placebo-controlled trial, 201 adolescents (ages 12–18 years) with obesity (a BMI in the 95^th^ percentile or higher) or with overweight (a BMI in the 85^th^ percentile or higher) and at least one weight-related coexisting condition were enrolled. Participants were randomly assigned in a 2:1 ratio to receive once-weekly subcutaneous semaglutide (at a dose of 2.4 mg) or placebo for 68 weeks, plus lifestyle intervention. The mean change in BMI from baseline to week 68 was − 16.1% with semaglutide and 0.6% with placebo (estimated difference, − 16.7 percentage points; 95% CI, − 20.3 to − 13.2; *P* < 0.001). At week 68, a total of 95 of 131 participants (73%) in the semaglutide group had weight loss of 5% or more, as compared with 11 of 62 participants (18%) in the placebo group (estimated odds ratio, 14.0; 95% CI, 6.3 to 31.0; *P* < 0.001). Reductions in body weight and improvement with respect to cardiometabolic risk factors (waist circumference and levels of glycated hemoglobin, lipids [except high-density lipoprotein cholesterol], and alanine aminotransferase) were greater with semaglutide than with placebo. The incidence of gastrointestinal adverse events was greater with semaglutide than with placebo (62% vs. 42%). This study assessed the efficacy and safety of once-weekly subcutaneous semaglutide plus lifestyle intervention among adolescents with obesity [135]. (LOE II-A)

*Bupropion* is a selective dopamine and norepinephrine reuptake inhibitor, while *naltrexone* is an opioid receptor antagonist. This combination is approved for obesity treatment in adults**,** off-label under 18 years [115]. (LOE VI-C)

*Setmelanotide* is a melanocortin-4 receptor (MC4R) agonist approved by the FDA for patients 6 years and older with obesity due to three rare genetic conditions: POMC deficiency, Proprotein Convertase Subtilisin/Kexin Type 1 (PCSK1) deficiency, or Leptin Receptor (LEPR) deficiency, confirmed by genetic testing demonstrating pathogenic, likely pathogenic, or of uncertain significance variants of these genes [136]. It is also used in clinical trials for other rare genetic disorders associated with obesity. These disorders include Bardet-Biedl Syndrome, Alstrom Syndrome, POMC and other MC4R pathway heterozygous deficiency obesity, and POMC epigenetic disorders [137]. (LOE I-A)

Several RCT studies to test new medications are ongoing and recently new molecules, i.e. dual agonists of GLP-1 and glucagon receptor, have been approved for adults with obesity. It is possible that in the future new medications will be available also for the use in children with obesity [138].

## Bariatric surgery

Bariatric surgery is the ultimate solution in adolescents with severe obesity, resistant to all other treatments, especially when serious complications are detected. (LOE VI-B)

In a large meta-analysis including 49 studies with 3,007 adolescents, the Roux-en-Y gastric bypass (RYGB) (*n* = 1,216), laparoscopic adjustable gastric banding (*n* = 1,028), and laparoscopic sleeve gastrectomy (*n* = 665) were the most common surgeries performed. At the longest follow-up (range 12–120 months), bariatric surgery led to an overall 16.43 kg/m^2^ and 31% reduction in BMI. After 12 months from surgery, there were significant improvements in glucose and lipid metabolism. The remission rate of dyslipidemia was 55%, 70%, and 95% at 1, 3, and > 5 years after surgery [139]. Preliminary data suggest sustained benefits after up to 9 years in terms of weight loss and very high remission rates for lipid parameters, uric acid, liver enzymes, prediabetes and diabetes [140].

The American Society for Metabolic and Bariatric Surgery Pediatric Committee and the American Academy of Pediatrics recently updated the recommendations for metabolic and bariatric surgery in youth, which removes the previous restriction on surgery based on pubertal or skeletal maturation [141, 142]. Current adolescent bariatric recommendations include BMI > 35 kg/m^2^ or 120% of the 95^th^ percentile for age and sex, with moderate to severe comorbidities (including obstructive sleep apnea (Apnoea Hypopnoea Index [AHI] > 5), type 2 diabetes mellitus, idiopathic intracranial hypertension, non-alcoholic steatohepatitis, slipped capital femoral epiphysis, Blount disease, gastroesophageal reflux disease and hypertension) or BMI > 40 kg/m^2^ or 140% of the 95^th^ percentile for age and sex [141, 142].

The authors state that previous barriers to surgery are not supported by evidence. According to guidance from the publications, no preoperative attempt at diet or exercise is necessary, a diagnosis of autism, developmental delay or syndromic obesity is not an automatic contraindication and unstable family environments, eating disorders, mental illness or prior trauma are not reasons to exclude surgery.

Contraindications to surgery are a medically treatable cause of obesity, untreated or poorly controlled substance abuse, current or planned pregnancy within 12–18 moths of the procedure, current eating disorder or medical, psychiatric, psychosocial or cognitive condition that prevents adherence to postoperative medication and lifestyle changes [141, 142]. (LOE VI-A)

There is no evidence to support the application of specific age limits for the timing of surgery [143, 144].

Surgery should be performed in a highly specialized center that guarantees an experienced multidisciplinary team. (LOE III-A)

Indication for surgery must be given on a case-by-case basis by the multidisciplinary team. (LOE VI-A)

However, there are inherent risks associated with bariatric surgery. In particular, post-operative complications, i.e. symptomatic gallstone disease and small bowel obstruction, may require further operative procedures. Furthermore, a previous study shows a slightly higher reoperation rate in the 5 years following RYGB in adolescents compared to adults (20–25%) [145]. However, recent improvements in operative technique and post-operative management have led to a reductions in the causes of reoperation after bariatric surgery [146].

Additionally, the risks of bariatric surgery include specific micronutrient deficiencies. Low iron and ferritin levels with result anemia were reported [143]; low vitamin D was documented insufficiency even at 5 years [147]; among patients with poor adherence to prescribed supplements, deficiencies in vitamins. A, B1, B6, and B12 and folate have also been described [143]. Adolescents have been shown to experience also substantial decreases in bone mineral density [148].

## Surgical options and results

Roux-en-Y gastric bypass is often referred to as the gold standard for surgical management of severe obesity in adults [149, 150] and adolescents [151] and is performed by using minimally invasive, laparoscopic surgical techniques. RYGB results in significant weight loss as a result of its effects on appetite, satiety, and regulation of energy balance [150].

Among all bariatric surgery options, vertical sleeve gastrectomy (VSG), is recommended in adolescents with severe obesity [143, 152]. Minimally invasive surgery, namely laparoscopic sleeve gastrectomy (LSG) is the most common performed bariatric surgery due to its safety, high efficacy and survival rates, in pediatric age. It represents a “vertical gastrectomy” performed on the greater curvature with preservation of the pylorus. Gastric tubulization by resection reduces approximately 80% of the stomach (remaining gastric capacity is > 100 mL). Operative time is also described to be shorter than the other surgeries. This technique achieves significant weight loss through similar effects on appetite, satiety, and regulation of energy balance and may reduce appetite through delayed gastric emptying and altered neurohormonal feedback mechanisms [153].

Compared to other approaches, the comorbidity resolution is maintained, weight loss efficacy is durable in children and adolescents > 5 years after surgery and specific nutrients’ malabsorption is reduced.

Nausea, dehydration, gastroesophageal reflux and wound infection (especially in patients with severe obesity), anastomotic leak and, rarely, gastric tube twist as well as gastric volvulus are reported in 4.3% of the cases and are considered as minor complications [154–156]. A retrospective analysis from the Metabolic and Bariatric Surgery Accreditation and Quality Improvement Program (MBASQIP) database (USA) showed that LSG was associated with a significantly lower rate of major complications in the first month after surgery [157–159]. (LOE III-B)

Laparoscopic adjustable gastric band (LAGB), a reversible procedure that accounted for approximately one third of all bariatric operations in the United States a decade ago [152], has experienced a significant decline in use among adults because of limited long-term effectiveness and higher-than-expected complication rates [160, 161]. Disappointing outcomes in the context of few prospective studies in the pediatric population have resulted in a similar decline in use of LAGB among adolescents [143]. At present, LAGB is limited by the United States FDA to people 18 years or older. (LOE IV-C)

Endoscopic sleeve gastroplasty (ESG) represents an attractive alternative to open surgery and is gaining popularity as first choice obesity operation. ESG is a bariatric endoluminal reversible procedure consisting in reducing and shortening the stomach by means of non-resorbable full-thickness sutures. It is performed endoscopically with an endoscopic suturing device and requires a learning plateau of 7–9 cases for experienced endoscopists. The plication starts on the anterior wall, 1 cm from the gastric incisura and proceeds all the way through the greater curvature to the gastric fundus which will be preserved. The gastric wall is pulled against the instrument, suctioned, in order to have a perpendicular full-thickness bite, and then, the stitch is released. Four to seven purse string stitches are enough to significantly reduce the gastric volume (< 100 ml) [162].

This technique has a positive impact on quality of life and it is considered to be a valid suggestion in adolescents or younger patients. Indication is reported in cases with lower BMI (30–35 kg/m^2^) or high risk patients or those patients who wish to undergo bariatric surgery. Storm et al*.* documented short- and long-term results on 1,607 ESG cases with a percentage of total body weight loss at 6, 12 and 18 months was 15.8, 17.1 and 17.3 respectively [163]. Success or failure of ESG seems to be predictable early after surgery and long-term gastroplasty integrity/weight loss are correlated with preoperative BMI. Comorbidities also seem to be treated. Patients get a global improvement of their physical (significant decreases in sistolic blood pressure, glycated hemoglobin, serum triglycerides and alanine transaminase [ALT]) and mental (improvements in self-esteem and lower rates of depression) health after ESG. Endoscopic and histopathologic evaluations showed no abnormalities within 1 year follow-up. Minor adverse events have been described (92%, nausea, vomiting, mild to moderate abdominal pain) and major problems (1.1–2%, fluid collections, late bleeding, refractory symptoms, intraoperative perforation, splenic injury and pulmonary embolism) [154]. ESG appears to be safe with the advantages of preserving the anatomy and being reversible and repeatable. However, the procedure still needs further assessment through a RCT to demonstrate efficacy in postoperative long term follow up in children and adolescents [146, 154, 164–166]. (LOE IV-B)

## Conclusion

Lifestyle modification therapy is the first necessary step of obesity treatment useful for improving many chronic disease risk factors and comorbidities but often fails to achieve clinically meaningful and sustainable weight loss. The addition of drugs to lifestyle therapy is the second step of treatment. Recently, new drugs approved for the use in adolescents were reported to be safe and effective in promoting remarkable weight loss.

The third level of treatment is bariatric surgery, that provided robust weight loss and risk factor/comorbidity improvements although potential risks have to be taken into account. Further research with long-term follow-up is necessary to assess durability of body weight improvement obtained with different treatments.

Obesity is not a homogeneous disease but is rather a complex, multifactorial condition that has multiple determinants that respond variably to a given intervention. Therefore, each mode of treatment employed in youths demonstrates considerable heterogeneity in response. Availability of predictors of the response to treatment, especially for weight-loss maintenance, could improve the success rate. New longitudinal studies will contribute to identify potential predictors as well as test the efficacy of pharmacotherapy in children younger than 12 years, for whom there are not yet drugs approved for treatment.

## Data Availability

Not applicable.

## Abbreviations

ALT: Alanine transaminase
AHI: Apnoea Hypopnoea Index
BMI: Body mass index
BT: Behavioral therapy
CBT: Cognitive behavioral therapy
CHO: Carbohydrate
CI: Confidence interval
DASH: Dietary Approach to Stop Hypertension
EMA: European Medicines Agency
ESG: Endoscopic sleeve gastroplasty
ETP: Therapeutic education of the patient
FDA: Food and Drug Administration
FFM: Fat free mass
FFQ: Food Frequency Questionnaire
GABA: Gamma-aminobutyric acid
GI: Glycemic index
GL: Glycemic load
GLP-1: Glucagon-like peptide 1
HOMA: Homeostasis model assessment
HOMA-IR: Homeostasis model assessment of insulin resistance
LAGB: Laparoscopic adjustable gastric band
LC: Low carbohydrate
LEPR: Leptin Receptor
LOE: Level of evidence
LSG: Laparoscopic sleeve gastrectomy
MBASQIP: Metabolic and Bariatric Surgery Accreditation and Quality Improvement Program
MC4R: Melanocortin-4 receptor
MD: Mediterranean diet
NAFLD: Non-alcoholic fatty liver disease
NICE: National Institute for Health and Care Excellence
PCSK1: Proprotein Convertase Subtilisin/Kexin Type 1
POMC: Pro-opiomelanocortin
RCT: Randomized control trial
RGL: Reduced glycemic load
RYGB: Roux-en-Y gastric bypass
VLCD: Very low-carbohydrate diet
VLED: Very low-energy diet
VSG: Vertical sleeve gastrectomy
WHO: World Health Organization
